# Supplementary material for: Dietary Habits and Psychological States during COVID-19 Home Isolation in Italian College Students: The Role of Physical Exercise
Source: Nutrients. 2020 Nov 28;12(12):3660. doi: 10.3390/nu12123660 (PMC7759990; doi:10.3390/nu12123660)
Supplement: Supplementary file 1 [file nutrients-12-03660-s001.pdf]

Table S1: Detailed descriptive statistics of the daily nutritional values, split for males and females.

Nutritional Variables	Males			Females		
	Q1	Median	Q3	Q1	Median	Q3
Energy (kcal)	1267.3	1672.5	2145.8	910.0	1197.0	1491.8
Protein (g)	60.4	81.4	110.7	43.8	59.6	79.1
Animal Protein (g)	20.4	37.3	57.8	12.7	27.0	43.3
Plant-based Protein (g)	13.6	22.2	33.3	9.4	15.7	23.7
Protein Value (g)	10.2	25.2	45.4	6.3	17.4	34.4
Lipids (g)	36.0	51.8	72.9	25.7	37.8	53.3
Saturated Fats (g)	9.9	16.4	25.3	6.9	11.8	18.4
Monounsaturated Fats (g)	9.1	15.6	24.0	6.2	11.3	17.6
Polyunsaturated Fats (g)	3.5	5.5	8.4	2.4	3.9	6.1
Animal Lipids (g)	10.4	20.8	36.6	6.9	14.9	24.6
Vegetal Lipids (g)	4.4	11.8	21.2	3.0	8.8	16.5
Omega-3 (g)	0.4	0.7	1.1	0.3	0.5	0.9
Omega-6 (g)	2.4	4.0	6.5	1.7	2.9	4.5
Carbohydrate (g)	149.9	206.5	271.4	103.4	143.5	189.1
Starch (g)	61.9	102.8	149.2	32.7	65.0	98.3
Glucides (g)	30.2	50.7	74.8	24.2	39.5	57.3
Glycaemic Index	47.8	55.0	63.8	47.6	54.5	63.2
Glycaemic Load	47.2	77.5	119.5	31.5	50.8	78.8
Soluble Fibre (g)	1.0	1.9	3.1	0.7	1.5	2.5
Insoluble Fibre (g)	3.0	6.0	10.4	2.6	5.2	9.0
Vitamin A (mcg)	303.6	683.2	1548.5	259.0	588.4	1199.0
Vitamin B6	1.0	1.5	2.1	0.7	1.1	1.6
Vitamin B12 (mcg)	0.7	2.3	4.9	0.3	1.5	3.5
Vitamin C (mg)	26.7	68.9	123.1	26.8	63.0	123.2
Vitamin D (mcg)	0.4	1.1	2.4	0.2	0.7	1.6
Vitamin E (mg)	3.1	5.3	8.0	2.3	4.2	6.4
Folic Acid (mcg)	134.2	209.2	304.2	112.2	182.4	273.5
Tyrosine (mg)	1080.2	1747.3	2627.2	733.9	1325.1	1937.8
Phenylalanine (mg)	1367.8	2216.2	3264.9	948.8	1656.3	2405.7
Tryptophan (mg)	328.1	555.2	827.5	220.9	405.4	614.6

Q1: first quartile; Q3: third quartile
